# Dialysis resource allocation in critical care: the impact of the COVID-19 pandemic and the promise of big data analytics

**DOI:** 10.3389/fneph.2023.1266967

**Published:** 2023-10-26

**Authors:** Farrukh M. Koraishy, Sandeep K. Mallipattu

**Affiliations:** Division of Nephrology, Department of Medicine, Stony Brook University Hospital, , Stony Brook, NY, United States

**Keywords:** critical care, resource deployment, dialysis, COVID-19, big data, artificial intelligence, machine learning

## Abstract

The COVID-19 pandemic resulted in an unprecedented burden on intensive care units (ICUs). With increased demands and limited supply, critical care resources, including dialysis machines, became scarce, leading to the undertaking of value-based cost-effectiveness analyses and the rationing of resources to deliver patient care of the highest quality. A high proportion of COVID-19 patients admitted to the ICU required dialysis, resulting in a major burden on resources such as dialysis machines, nursing staff, technicians, and consumables such as dialysis filters and solutions and anticoagulation medications. Artificial intelligence (AI)-based big data analytics are now being utilized in multiple data-driven healthcare services, including the optimization of healthcare system utilization. Numerous factors can impact dialysis resource allocation to critically ill patients, especially during public health emergencies, but currently, resource allocation is determined using a small number of traditional factors. Smart analytics that take into account all the relevant healthcare information in the hospital system and patient outcomes can lead to improved resource allocation, cost-effectiveness, and quality of care. In this review, we discuss dialysis resource utilization in critical care, the impact of the COVID-19 pandemic, and how AI can improve resource utilization in future public health emergencies. Research in this area should be an important priority.

## 1 Introduction

In the United States, the cost of an intensive care unit (ICU) stay is approximately $4,300 per day, and the total annual critical care cost is estimated to be approximately $108 billion, which is 13.2% of total hospital costs and 4.1% of the national health expenditure (1). The past decade has seen an increasing demand for critical care due to longer life expectancy, greater comorbidity burden, and better survival rates in the ICU (1–4). “Access to critical care” is defined based on the patient’s capacity to benefit from the available resources (5). Often the sickest patients admitted to the ICU have the highest capacity to benefit in terms of reduction in mortality risk, making admissions of these patients more cost-effective (measured by the cost per life-year saved) than those of patients with a lower risk of death (6). Due to the increasing demands and limited resources and budgets, critical care has become a scarce resource, leading to the undertaking of value-based cost-effectiveness analysis (CEA) and resource rationing to achieve the most efficient and effective patient care (2).

The resource utilization policies in the ICU should be guided not just by resources, but also by the goals of equity of access and prevention of both short- and long-term morbidity and mortality (2). Although clinician judgment can be effective at identifying the patients who would benefit most, very large volumes of valuable real-time ICU data are underutilized. Artificial intelligence (AI)-based data analytics are being utilized in multiple data-driven healthcare services such as decision support (7–9), prediction of disease trends and outcomes (10), and long-term health (11). AI also has tremendous potential to improve the optimization of healthcare system utilization (12) through the analysis of extremely large datasets. In this review, we will discuss how AI can improve the utilization of scarce critical care resources, using the lessons learned from dialysis utilization during the COVID-19 pandemic.

## 2 Dialysis resource management in critical care

### 2.1 Utilization of dialysis in critically ill patients

Dialysis is one of the most common procedures utilized in the ICU (13). Although neither of the two most commonly used modalities—continuous kidney replacement therapy (CKRT) and intermittent hemodialysis (IHD)—offers a significant survival advantage over the other (14), IHD is the more common modality of choice, and CKRT is typically recommended for patients with hemodynamic instability and those with high levels of intracranial pressure (15, 16). CKRT is also used in fluid management (especially when the obligate intake is large), burns, sepsis, and heart and liver failure patients (17). The choice of modality has been variable and is based on local ICU practices and the availability of resources (18). CKRT is more costly than IHD. In a base-case analysis, CKRT was found to cost $3,679 more than IHD per patient (19). In addition to machine cost, there is a significant cost associated with the large volume of dialysis and replacement fluids involved in CKRT (20). In addition, whereas CKRT is typically conducted by the ICU nurse taking care of the patient, IHD requires dialysis nurses, which adds to the personnel cost (21). Several studies have looked at the cost-effectiveness of CKRT compared with IHD (20, 22–24). In a *post-hoc* analysis of the BEST Kidney Study, using data from 53 centers across 23 countries, Srisawat et al. reported that the median cost per day was $289.60 (IQR $830.8–$116.8) greater for CKRT than for IHD, and that reducing the replacement fluid volumes increased the cost savings (20). In another study of critically ill patients with AKI, despite the greater initial cost of CKRT in the ICU, the 5-year total cost, including the cost of dialysis dependence, was lower for CKRT than for IHD (24). In a recent systemic review of seven published CEAs of CKRT vs. IHD, Singh et al. reported a marginal quality-adjusted life year (QALY) gain for CKRT, and that the cost-effectiveness of CKRT was mainly due to the long-term dialysis dependence rates (23).

### 2.2 Cost estimation for dialysis utilization in the ICU

Cost estimation in critical care is confounded by other factors such as pre- and post-ICU healthcare and the clinical diversity of patients, making the average daily ICU cost variable from one patient to another (2). Minimizing ICU costs can potentially be plagued by “cost shifting”, that is, greater costs incurred in the provision of non-ICU health services for the same patient. Unfortunately, most of the earlier published CEAs in critical care have focused on short-term outcomes such as ICU mortality and length of stay. However, the estimation of long-term outcomes, such as Health-Related Quality of Life (HRQOL) (25), socioeconomic impact of critical illness (26), and measures of longer-term morbidity and mortality in ICU survivors are crucial to more accurately determining cost-effectiveness and guiding decision-making on resource utilization (2). Patient-centered outcomes and societal perspectives are very important to improving the quality of CEA (27–29). In a study of 959 patients with AKI who were treated with dialysis in the ICU and followed up for 3 years, the main factors found to be associated with major adverse kidney events (death, incomplete kidney recovery, or development of end-stage kidney disease [ESKD]) were severity of illness and use of CKRT (vs. IHD) during the ICU admission (30). In another study looking at ICU survivors, the QOL, although lower at 3 months post-discharge, was similar at the 1- and 4-year follow-ups among those admitted to the ICU with acute kidney injury (AKI) requiring dialysis and those without AKI (31). Therefore, both short- and long-term healthcare expenditure and outcomes in critically ill patients with AKI should be analyzed for robust cost estimation. The factors traditionally used in the allocation of dialysis are summarized in **Table 1**.

**Table 1:** Factors traditionally linked to the cost of dialysis in the ICU.

Dialysis-related costs	Staff-related costs	Hospital-related costs	Patient-related costs
Dialysis machines	ICU nurse vs. dialysis nurse	ICU beds	Dialysis access placement
Dialysis solutions	Nursing time	Patient monitoring devices	Need for large dialysis volumes and adequate clearance
Dialysis filters (type and life span) and dialysis tubing	Technician time	Patient and machine transport	Hypercoagulable state
Anticoagulation medications	Clinician time		Need for vasopressors during dialysis-related hypotension
Other intravenous solutions such as calcium and saline			

ICU, intensive care unit.

## 3 Impact of the COVID-19 pandemic on dialysis allocation

### 3.1 Critical care resource allocation during the COVID-19 pandemic

Healthcare emergencies such as natural disasters and infectious disease pandemics exert a severe impact on critical care resources and require the creation of comprehensive policies on resource allocation (32). The influenza pandemics at the start of the 21st century, such as the H5N1 (“bird flu”) and H1N1 (“swine flu”) pandemics, resulted in acutely limited availability of mechanical ventilators (33), and triage protocols for resource deployment based on the Sequential Organ Failure Assessment (SOFA) score (34) were created to allocate access to critical care resources (35, 36). However, critical care triage during pandemics simply based on physiological scores such as SOFA has its limitations, and protocols should be modified based on other data, such as those related to outcomes, as they emerge in real-time (36).

The coronavirus disease 2019 (COVID-19) (37) pandemic resulted in an unprecedented burden on critical care units, leading to the reassessment of the existing pandemic utilization and allocation policies to better prepare ICUs for the unique challenges posed by COVID-19 (38). At the start of the pandemic in the United States, an Expert Panel Report of the Task Force for Mass Critical Care and the American College of Chest Physicians recommended a triage system to guide critical care resource management toward the patients who were most likely to benefit while maintaining the standard of care (32). Over the last 3 years, the emergence of new variants, variable isolation protocols, mass vaccination policies across nations and regions, and evolving therapies have led to difficulties in enforcing resource allocation policies that need to be constantly adapted in line with the changing face of the COVID-19 pandemic (39). This has led to the recognition of the importance of utilizing large real-time datasets for institutional planning and resource allocation to ensure standards of care under crisis and to meet the egalitarian and utilitarian principles of clinical practice.

### 3.2 Allocation of dialysis during the COVID-19 pandemic

AKI is common in patients with COVID-19 (40, 41) and is associated with in-hospital complications and mortality (41–47). A high proportion of COVID-19 patients admitted to the ICU, especially in the first year of the pandemic, required CKRT (48–50), resulting in a major burden on critical care resources. Assessing the risk of respiratory failure and the need for ventilators were not immediately recognized as priorities when treating patients with COVID-19, and similarly, the impact of dialysis-requiring AKI was not fully understood until later in the pandemic (51, 52). In addition to dialysis machines, there was a major increase in demand for all components of hospital dialysis programs, including nursing staff, technicians, and consumables such as dialysis filters and solutions and anticoagulation medications (53). In addition to patients who developed AKI, dialysis requirements also increased for patients with ESKD, who also had significantly increased rates of ICU admission (53).

This crisis led to the development of resource management policies in acute dialysis units across hospitals (53). The shortage of CKRT machines led to the creation of regional and institutional policies on increasing supply and developing stockpiles of CKRT machines (54, 55). Some academic centers adopted crisis protocols of prolonged intermittent kidney replacement therapy (56) or accelerated venovenous hemofiltration (57), and CKRT devices could be shared between patients. This novel strategy entailing the sharing of tools among patients undergoing CKRT in ICUs in close geographical proximity reduced nursing workloads and the need for machine transportation (58). However, the sharing protocols increased the demands of dialysis fluids due to the increased flow rates required for effective clearance. To address this demand, some centers adopted the policy of “fixed hours” on CKRT (51). Alternate dialysis strategies such as sustained low efficiency dialysis (SLED) that utilize the hospital water supply, hence minimizing the need for consumable dialysis solutions, were adopted in some centers (59). However, the drawback with SLED was the requirement of a dialysis nurse since most ICU nurses are not trained in this modality. To address the issue of nursing staff shortage, at our institute we adopted the remote telemonitoring of dialysis treatments, which was found to be safe, reliable, and minimized the risk of staff exposure (60). Some hospitals also employed acute peritoneal dialysis (APD) to expand their capacity and to tackle the shortage of hemodialysis machines (61). However, the role of APD in critically ill patients with COVID-19-related respiratory failure is limited by the risks of ventilatory compromise, especially in patients with requirements for high levels of oxygen, positive end-expiratory pressures, or proning (61, 62). The increased hypercoagulability seen in COVID-19 patients posed another challenge, due to the reduced filter life span and constant need to change filters, leading to increased costs and nursing workload (63). This led to the enforcement of anticoagulation protocols to minimize filter clotting. There is a dearth of data on CEAs in critical care dialysis allocation during the COVID-19 pandemic. One study of patients with ESKD comparing dialysis modality found that IHD was associated with a greater cost than PD during the COVID-19 pandemic (64). Finally, another challenge was the arrangement of outpatient dialysis centers, especially for new patients who required long-term dialysis. The development of isolation protocols and the arrangement of isolation rooms in chronic dialysis units with limited resources did not occur at a rate equal to the rapid increase in demand. This led to prolonged hospitalizations.

In summary, a large number of factors are involved in dialysis resource allocation to critically ill patients, especially during public health emergencies (**Table 2**). Using large datasets and smart analytics that can take into account all the relevant healthcare information in the hospital system could lead to efficient and cost-effective dialysis resource allocation in future public health emergencies.

**Table 2:** Non-traditional data can be utilized for AI-based dialysis resource allocation and CEAs in critically ill patients during healthcare emergencies.

Dialysis/staff	Hospital/administration	Patient	Outcomes (short- and long-term)
Hemodynamic data	ICU budget	Primary diagnosis	ICU mortality
Ultrafiltration data	Geolocalization of dialysis services	Demographics and zip codes	Hospital mortality
Blood pressure medications	Back-up resource generation	Comorbid medical/mental conditions and pain	Long-term mortality
Stockpiles and inventory of dialysis machines and dialysis catheters	Emergency policy committees	Severity of illness scores	Length of ICU stay
Backup dialysis staff	Redeployment protocols	Medications (outpatient and inpatient)	Length of hospital stay
Backup ICU staff	ICU rooms	Laboratory and other tests (outpatient and inpatient)	Hospital readmission rates
Staff training	Weekend coverage	Baseline ESKD and years on dialysis	Duration of acute dialysis requirement
Social distancing and contact tracking	Isolation rooms	Ventilator and vasopressor data	Days to recovery from AKI
Return-to-work policies	Telemedicine services	Nutritional and functional status	Inpatient nephrology follow-up
PPE	Crisis teams	Long-term dialysis access	Post-AKI CKD
Utilization of APD and SLED	Daily census	Infections, antibiotics, and vaccination data	Post-AKI ESKD
Data on machine sharing and reduced dialysis flow rates/frequency/time	Inventory and shipment tracking	Transplant status	HRQOL
Anticoagulant dosing	Pharmacy, radiology, and vascular surgery services	Residence location, education level, employment, and socioeconomic status	Outpatient nephrology follow-up
Dialysis prescription		Substance abuse	
Missed and/or incomplete dialysis sessions		Imaging data	
Dialysis refusal/withdrawal		Resuscitation data	
Staff feedback		Patient feedback	
		Wearable devices	
		Health literacy	
		Health insurance	
		Research data	

ICU, intensive care unit; AKI, acute kidney injury; ESKD, end-stage kidney disease; HRQOL, health-related quality of life; PPE, personal protective equipment; SLED, slow low-efficiency dialysis; APD, acute peritoneal dialysis.

## 4 The role of big data and AI in optimizing utilization and allocation of dialysis resources

### 4.1 Overview of big data and AI in healthcare

The use of advanced information technologies to improve healthcare has seen a rapid advancement since the turn of the millennium owing to the rapid progress made in computer science and information technology (65). Modern health data analytics is focused on high-quality, efficient, and cost-effective care delivery and personalized medicine (66–70). This requires the ability to capture, integrate, and analyze heterogeneous unstructured and structured data in real-time from healthcare systems (71). Healthcare systems such as ICUs generate very large volumes of data constantly. The rapidly evolving AI techniques such as machine learning (ML) have the ability to analyze extremely large datasets, learn patterns, and identify non-linear associations and causal relationships (72, 73).

Healthcare data analytics include “data-driven” methods that include ML and pattern recognition algorithms and are categorized as supervised (classification) and unsupervised (clustering) learning models (74). “Knowledge-driven” methods include logic-based knowledge modeling and reasoning algorithms based on clinical data (75) and can be more readily applied to clinical decision-making (72). The applications of these models include “decision analytics” (e.g., the diagnosis and treatment of diseases), “predictive analytics” (e.g., using real-time data to predict health outcomes), “prescriptive analytics” (e.g., the simulation of future outcomes), “comparative analytics” (e.g., using data to compare impact of two different interventions on an outcome), and “semantic analytics” (e.g., using large datasets to understand complex relationships between the variables for hypothesis generation and testing) (72).

As the volume of data provided by ICUs and complexity of healthcare data have increased tremendously, there is an increasing need to develop general-purpose frameworks that allow non-experts to be able to easily apply complex AI algorithms to health management (72). It is important that non-AI experts understand the various facets of healthcare data, such as “variety” (multiple novel data sources such as biometrics and “omics” data), “veracity” (source and accuracy of data), “volume” (amount of data), “velocity” (the rate at which new data are generated), and, finally, “value” (knowledge gained and potential for clinical application) (76). All these data features are typically high in the ICU, making it an ideal setting for intelligent data analytics. However, to apply the correct analytical methods and to properly interpret the analysis, a proper understanding of each of these facets of healthcare data is critical.

### 4.2 The use of big data and AI in critical care resource allocation

Data mining and AI technology are now being increasingly used for human resource management systems and to improve workflows and cost–benefit ratios in healthcare systems (77–79). Previous studies have shown the utility of AI in the efficient scheduling of operating rooms (80), improvement of patient waiting times and staff workflow (81–84), reduction of hospital discharge time and length of stay (85), efficient hospital bed allocation (86), and the reduction of response time and hospital costs (87).

AI and big data analytics have great applications in mobile (“m”)-health (88) and in the recent pandemic, the use of tele-ICU in care delivery has greatly increased, especially by hospitals in remote, underserved settings (89). An intelligent remote monitoring system with innovative data analytics has been developed for the post-ICU monitoring of patients with COVID-19, which has the potential to reduce the length of ICU stays (90).

The unprecedented challenge created by the COVID-19 pandemic and the constant emergence of new data has further highlighted the importance of the integration of smart advanced information technologies in healthcare (91). Sottile et al. recently reported the use of smart analytics to predict mortality in patients hospitalized with COVID-19 using electronic health record (EHR) data (92). Cheng et al. reported the use of AI-based tools to predict the risk of ICU transfer in patients admitted for COVID-19 (93).

Therefore, the use of big data and AI-based technology has great potential to improve the management and allocation of resources in critical care.

### 4.3 The use of big data and AI in the allocation of dialysis

There are many factors to consider in the management of resources related to dialysis utilization. In one study conducted among hospitalized patients requiring dialysis, factors such as health insurance, ischemic heart disease, late referral to the nephrologist, and the use of temporary vascular access for the first dialysis were identified as the major factors contributing to an increased length of hospital stay (94). Other studies have identified that poorly controlled anemia, depression, and pain were factors associated with the increased utilization of hospital dialysis resources (95, 96). Limited health literacy was shown to significantly increase the risk of hospitalization among patients on chronic dialysis (97). As mentioned, the allocation of dialysis resources was a major challenge during the COVID-19 pandemic that required the development of emergency programs (98). The shortage of resources also resulted in ethical challenges for patients, families, clinicians, and policymakers for resource prioritization (99, 100).

To ensure equity and justice, there is a critical need to harness big data to maximize the cost-effective utilization of dialysis resources while maintaining the standard of care in critically ill patients. Although previous studies have identified important factors that can be addressed to improve the management of dialysis resources (51, 54–59, 61–63, 94–100), the availability of large volumes of EHR data in dialysis patients is a prime resource for the application of AI-based smart analytic tools for resource management (**Table 2**). The use of AI-based data analytics is rapidly increasing in the field of nephrology, and its application has increased our understanding of disease pathogenesis, outcome prediction, and the personalization of medicine (101). ML-based analytic models have been shown to outperform clinician-based predictions related to AKI (102–104). We have previously shown the use of X-Boost-based machine learning algorithms to predict the severity of AKI, the need for dialysis, and renal recovery in patients with COVID-19 (105). Data from existing repositories such as the EHR can help clinicians and administrators better triage existing dialysis resources to the hospitals that are and the patients who are predicted to need them most. Future research in this area should be an important priority.

### 4.4 Knowledge gaps and challenges in the use of big data and AI

As with any innovative technology, the hype and expectations surrounding intelligent data analytics have to be modified based on their limitations and drawbacks. The incorrect selection of datasets or analytic tools, erroneous processing of data, and incorrect interpretations of the results are major challenges that will need to be overcome in a field such as healthcare where the health of patients is at stake (**Table 3**). In a systemic review of 46 studies of the use of ML models to predict AKI, a high risk of bias and lack of external validation of the models was observed (102).

**Table 3:** Key factors in the application of AI-based analytics of EHR data for dialysis resource allocation.

Correct selection of datasets or analytic tools
Correct data processing
Rigorous data quality checks
Harmonization of data from different EHR systems
Data security
Correct interpretations of the results
Minimize bias
External validation of tools
Output data needs to be easily understood by clinicians (XAI)
Patient education
Patient privacy and confidentiality
Resource supply and demand monitoring
Incorporation of evolving disease management guidelines, especially during new health emergencies such as epidemics/pandemics
Cyber security
Development of regulations and guidelines for the use of AI-based smart analytics in patient care

AI artificial intelligence; XAI, explainable artificial intelligence; EHR, electronic health record.

To develop the trust of the healthcare community in this technology, the output data need to be clinically explainable and the processes transparent (106). In addition to clinicians, it will also be important to develop patient trust; hence, patient education will be crucial. Explainable artificial intelligence (XAI) is the next frontier in AI-based healthcare analytics (107). As with all aspects of healthcare delivery, patient privacy and confidentiality are paramount (108) and the privacy risks should be communicated to the patients and their consent obtained (109). Several regulatory compliance steps for data security might limit the use the AI systems until transparency and understanding improve (110). Patient characteristics, the nature of diseases, and the types of diagnostic tests and treatments can change with time, and this needs to be accounted for when updating existing AI tools and developing new ones. Rigorous checks on data quality must be carried out, and the harmonization of data across different EHRs in different health systems should be conducted before their application for analysis. In our recent study on characterizing AKI in patients with COVID-19, we leveraged EHR data in patients with COVID-19 from 53 healthcare systems across the United States (41). State-of-the-art data cleaning, quality control, and harmonization processes were undertaken before the analytical steps (41). Finally, the vulnerabilities of AI models to “adversarial attacks” should be routinely monitored and appropriate precautionary and corrective measures should be applied (111).

The development of laws, regulations, and guidelines for the use of healthcare data and AI-based smart analytics needs to be developed for use in all aspects of healthcare, including resource allocation (112). Improved accuracy of the data and understanding of AI tools would strengthen the healthcare community’s trust in them (113).

## 5 Conclusions

The COVID-19 pandemic resulted in an unprecedented burden on critical care units worldwide. Dialysis machines became scarce, leading to value-based CEA and the rationing of resources to deliver patient care of the best quality. AI-based big data analytics are now being utilized in multiple data-driven healthcare services, including the optimization of healthcare system utilization. Data from existing repositories such as the EHR can help clinicians and administrators to better triage existing dialysis resources to the hospitals and patients who are predicted to need them most. Although the application of AI-based big data analytics for dialysis allocation in critical care holds a lot of promise, several challenges need to be taken into consideration. Future research in this area should be an important priority.

## Author contributions

FK: Conceptualization, Funding acquisition, Investigation, Methodology, Resources, Validation, Visualization, Writing - original draft, Writing - review & editing. SM: Conceptualization, Funding acquisition, Resources, Validation, Visualization, Writing - review & editing.
